# Contrasting a Cassian-Merton model of mystical prayer with moments of Big-C creativity in contemporary music

**DOI:** 10.3389/fpsyg.2024.1388789

**Published:** 2024-11-21

**Authors:** David Priilaid, Christian Callaghan

**Affiliations:** ^1^School of Management Studies, University of Cape Town, Cape Town, South Africa; ^2^School of Management, Anglia Ruskin University, Cambridge, United Kingdom

**Keywords:** creativity, mysticism, prayer, music, interdiscipinary

## Abstract

Within the psychology literature there is a dearth in understanding how and why instances of heightened creativity, or ‘Big-C’, may manifest randomly, often at an early age with minimal practice and experience. In contrast to ordinary “little-c” creativity, depictions of profound creative moments and the means by which they can be attained therefore remain as enigmatic as acts of mystical prayer. Pursuing this metaphor: with little-p equating to ordinary prayer and Big-P equating to mystical prayer, from the work of St. John Cassian and Thomas Merton, we contrast the developmental path of prayer with subjective accounts of little-c and Big-C moments of creativity in the lives of famous 20th century Anglophone artist-musicians. Here, we identify a symmetry between the aetiology of prayer and creativity. As with little-p, the development of little-c creativity is sequential, tracing a path from *authenticity* to *task motivation*, to *practice* and then *experience*. By contrast, akin to Big-P, Big-C creativity may be obtained moving from *authenticity* to *task motivation*, but thereafter circumventing the requirements of elevated *practice* and *experience*. Moreover, in moments of extreme creativity, artists manifest a profound surrender of agency as Big-C emerges spontaneously with minimal deliberate thought and an absence of ownership. Thus, drawing on the insights of Cassian-Merton, we present a historically grounded perspective on the spontaneous and enigmatic emergence of Big-C creativity.

## 1 Introduction

*The slenderest knowledge that may be obtained of the highest things is more desirable than the most certain knowledge obtained of lesser things.*

Saint Thomas Aquinas, *Summa Theologica I q. 1 a. 5* AD 1.

*Silence, simplicity and humility… the only proper state for the artist as for the human being.*

Patrick White, *The Vivisector.*

The field of psychology is instrumental in exploring creativity, especially in understanding the process of idea generation. Here a fundamental framework revolves around the twin constructs of Big-C and little-C (Kozbelt et al., 2010). Big-C represents the realm of ground-breaking ideas that have the power to revolutionize the world. Kaufman and Beghetto (2009) categorize instances of Big-C as exceptional, unequivocal displays of genius-level creativity, while Feldman et al. (1994, p. 1) define it as “the attainment of something extraordinary and innovative, something capable of transforming and significantly altering a particular field of endeavor.” In contrast, little-C encompasses the realm of everyday, commonplace creativity that arises from routine practice (Merrotsy, 2013). Although these two terms are conventionally considered as opposing concepts, little-C has since been expanded by Kaufman and Beghetto (2009) to encompass three distinct sub-types: the creative activities of children, known as mini-C; the endeavours of amateur artists, referred to as little-c; and the polished, professional work of seasoned creative experts, termed Pro-c.

In light of these distinct categories of creativity and the corresponding discussions and elucidations provided by Beghetto and Kaufman (2007); Kaufman and Beghetto (2009), the central question shifts from mere delineation within this four-part typology, turning instead to the essence of what enables Big-C creativity to manifest. In essence, to echo Lubart’s inquiry (Lubart, 2001), what is it, exactly then, that makes Big-C so creative?

It is intriguing to note that among the many brilliant artists and thinkers who have reached the pinnacles of Big-C creativity, none of them seem to grasp the precise process that leads to such moments. The stark reality is that most individuals often operate within the shallows of little-c creativity, only occasionally, and sometimes seemingly by chance, venturing into the realm of genuine creative brilliance. The question that arises is: Why is this the case? While many can attain moments within the lower limbs of the creative architecture, few attain the upper reaches, and if they do, most have little or no clue how they got there, and on their return to little-c, declare themselves uncertain as to whether they can attain such creative heights again.

As the singer-songwriter Leonard Cohen (2011) wryly observed on receiving the Prince of Asturias poetry award: “Poetry comes from a place that no one commands, that no one conquers. So I feel somewhat like a charlatan to accept an award for an activity which I do not command. In other words, if I knew where the good songs came from I would go there more often.” In 1969, Paul Simon’s composition of “Bridge over Troubled Water” flowed so effortlessly that he later pondered, “Where did that come from? It does not sound like me” (Eliot, 2010, p. 105). Later, on separating from Art Garfunkel, Simon was so unsure of his song writing abilities that he took song writing lessons. Similarly, the songwriter, Jimmy Webb, was living in the back of his car when he wrote the Grammy winning “Up, Up and Away” in 1967 for the 5th Dimension. Later, on finding success with songs covered by Glen Campbell, he bought a fantastic house, added a recording studio, and then found he could not write. By the close of the decade, Webb’s magic touch had completely disappeared (Rachel, 2013).

Faced with such anecdotal evidence, existing models of creativity development (see for example: Amabile, 1983; Fürst et al., 2017; Kozbelt et al., 2010; Nijstad and Stroebe, 2006; Priilaid and Callaghan, 2023; Simonton, 2014; Torrance, 1988, 1995; Watts et al., 2019) have little to say about how moments of Big-C creativity emerge and then disappear. Here, one of the major confounding issues is why moments of creative genius may occur in contexts of little artistic experience and practice. This contradicts intuitive expectations that creativity should correlate inherently with degrees of experience, and practice: which is to say that given sufficient perseverance, Big-C events should inevitably occur and then re-occur. Yet more often than not, Big-C creativity remains beyond the reach of mere domain-specific experts (Simonton, 2000). If anything, moments of Big-C creativity track a hit-and-miss trajectory, with an aetiology that even great artists struggle to explain.

Recognizing the limitations of current theories in elucidating the nature of Big-C creativity, we commence this study with the assertion that it remains an enigmatic and inadequately explained phenomenon—an intellectual “black box,” so to speak. Consequently, we position moments of Big-C within the frontier-realm of paranormal psychology, drawing a parallel with a profoundly ancient spiritual framework, that of Mysticism, and more specifically, mystical prayer. Building upon the foundational concepts laid out by St. John Cassian over 1,600 years ago, we put forth a comprehensive theory of mystical manifestation further developed by the erudite Trappist monk, Thomas Merton. This theory, which we term the Cassian-Merton Theory of Mystical Prayer, offers a more detailed and contemporary perspective on the mystical experience.

So doing, with little-p and Big-P, respectively, denoting ordinary prayer and mystical prayer, we compare this “spiritual” theory to moments of little-C and Big-C creativity as subjectively defined by major icon-level artists spanning the singer-songwriting era of the 1960s, 1970s, and 1980s. Here our aim is to show how Cassian-Merton applies also in the development of Big-C creativity.

In pursuing this endeavour, we aim to contribute to the creativity literature across several overarching domains:

1. *Rediscovery and formalization of the Cassian-Merton model*: Previously hidden in obscure 4th-century Latin texts originally authored by St. John Cassian and briefly alluded to in a 2012 audio-book containing some of Merton’s lectures, we now identify, formalize, and establish a direct connection between the Cassian-Merton model and the body of literature central to creative development. This synthesis draws from the works of influential scholars such as Acar and Runco (2019), Amabile (1983), Andreasen (1987), Baas et al. (2016), Lubart (2001), and Simonton (2000).
2. *Mystical prayer as a counterpart to flow-driven creativity*: Building on this foundation, we propose that the non-sequential model of mystical prayer stands in stark contrast to process-oriented conceptions of conventional prayer. In its depiction of how moments of mystical prayer may manifest in the relatively young and inexperienced, it exhibits a strong symmetry with explanations of how states of flow lead to the emergence of Big-C creativity, aligning with the insights of Nakamura and Csikszentmihalyi (2002).
3. *Biographical analysis of late-20th century Anglophone music culture*: Our research endeavours extend to a more comprehensive understanding and modelling of the highly creative contributions within the late-20th-century Anglophone musical culture. In doing so, we contribute to the tradition of biographical analysis in the creativity literature, drawing inspiration from the works of Andreasen (1987), Dávila and Ruiz (2019), Hadamard (1945), Ludwig (1992), Post (1994), Priilaid and Callaghan (2023), and Rossman (1931). We recognize that this cohort exclusively comprises successful musicians, acknowledging the variances found within cross-cultural groupings, as demonstrated by research from Benedek et al. (2014) and Sica et al. (1997).
4. *Positioning Big-P and Big-C within the evolution of modern Mysticism*: By aligning Big-P and Big-C as interconnected facets originating from the same mystical source, following Schmidt’s (2003) two-stage aetiology of modern Mysticism, we identify Big-C as a third stage in its evolution. While these contributions are transdisciplinary, they are rooted in the field of psychology, as best as it advances our understanding of behaviour, cognition and the intricacies of the human condition.

## 2 Materials and methods

The approach of this study is as follows. After reviewing the mystical and prayer-related literature, we introduce the Cassian-Merton model. This model distinguishes between mystical prayer, termed Big-P, and regular prayer, denoted as little-p. We uncover the distinctive attributes of little-p, which follows a sequential developmental path, contrasting with the non-sequential trajectory of Big-P. To enhance clarity, we depict this model diagrammatically, emphasizing the divergent dimensions of these two prayer forms.

With this groundwork laid, we outline and incorporate biographical material from the creative lives of certain music luminaries from the 1960s, 1970s, and 1980s to demonstrate how moments of Big-C development echo the Cassian-Merton descriptions of Big-P. Included here are the singer songwriters: Bob Dylan, Neil Young and Leonard Cohen; the ‘Rock ‘n Rollers’ Keith Richards and Bruce Springsteen, and the jazz-driven musicians Donald Fagen and Bill Evans. While open to critiques of subjectivity, lack of verifiability and risk of overgeneralization, biographic analysis (viz: Andreasen, 1987; Dávila and Ruiz, 2019; Ferrarotti, 2005) provides a useful methodology when the objective of study is to derive personal insight, contextual richness and emotional connection in understanding an individual’s behaviour and career progression.

Our subsequent model of creative development unveils a fusion of stages, encompassing both sequential and non-sequential phases, mirroring the principles inherent in the Cassian-Merton framework. These intricate dynamics have, until now, received insufficient attention within existing theories on creativity. Consequently, our objective here is to delineate and elucidate the origins of both Big-C and little-c variants of creativity, with the aim of addressing the fundamental questions of “what causes what, and why, and how the outcome of this causal mechanism varies depending on the circumstances” (Carlile and Christensen, 2005, p. 16).

## 3 Mysticism

Before introducing the work of Merton, a foregrounding of the concept of Mysticism and its historical development is appropriate. Writing for a post-Cold-War world; in the year before his coronation as Pope of the Catholic Church, the then Cardinal Joseph Ratzinger acknowledged the ongoing pull of Mysticism as portent of an alternate realm, observing that:

In the leaden loneliness of a God-forsaken world, in its interior boredom, *the search for mysticism*, for any sort of contact with the divine has sprung up anew. Everywhere there is talk about visions and messages from *the other world*, and wherever there is a report of an apparition, thousands travel there, in order to discover perhaps, *a crack in the world*, through which heaven might look down on them and send them consolation (Ratzinger, 2004, p. 487, emphasis added).

To expand on Ratzinger; it is evident that Mysticism encompasses a broad spectrum of beliefs, practices, and experiences aimed at establishing direct communion with profound spiritual truths and the transcendent divine. This pursuit integrates deeply intuitive encounters facilitated through techniques such as meditation, contemplation, ritual, and inner exploration, and it is a common thread woven throughout various religious traditions.

Spanning Christianity, Islam, Judaism, Hinduism, and Buddhism, mystics across history have consistently reported ineffable encounters with unity, transcendence, and an overwhelming sense of oneness with the universe or a higher spiritual reality. Mysticism has exerted a profound influence on the evolution of religious and philosophical thought over the ages and continues to be a subject of interest and academic inquiry within the realms of theology, philosophy, comparative religion (Schmidt, 2003) and the ongoing interest in mapping AI’s comparative characteristics compared to that of human consciousness (Dehaene et al., 2021; Settecase, 2024).

### 3.1 The theological concept of mystical prayer

The study of prayer holds a peripheral position within the scientific academy, largely due to challenges in achieving replicable results and a prevailing view that metaphysical subjects extend beyond empirical investigation. Nonetheless, within theological epistemology, prayer, and particularly mystical prayer, remains a focal subject. Mystical prayer is regarded as a practice dedicated to fostering receptive silence, inner stillness, and a profound sense of unity with the divine, rather than verbal requests or communication. This form of prayer, aimed at transcending ordinary thought through deep spiritual connection, is integral to various religious traditions, including Zen meditation, Christian Centering Prayer, Islamic Sufi Dhikr, and Hindu Bhakti Yoga.

Within the last century, prominent scholars and their works related to mystical prayer include: the Harvard University psychologist and philosopher, James, and his classic work on different types of mystical experiences, “*The* Var*ieties of Religious Experience*” (James, 1902); Underhill, and her foundational text: “*Mysticism: A Study in the Nature and Development of Spiritual Consciousness*” (Underhill, 1910), which examined mystical practices across different religious traditions, and McGinn’s “*The Foundations of Mysticism: Origins to the Fifth Century”* (McGinn, 1991).

Allied to these formative writings of the modern era, Mysticism has been explored through the Buddhist concept of mindfulness by mostly non-religious writers and practitioners such as Sam Harris, Tara Brach, Mark Epstein, Jon Kabat-Zinn, and Eckhart Tolle. A number of academics have also explored non-religious approaches to contemplative practices, including aspects of mystical prayer; doing much to bridge the gap between traditional spiritual practices and modern secular contexts. Within this cohort are included: the psychologists Ronald Siegel, Rick Hanson, Kristin Neff and Christopher Germer; the neuroscientist Shinzen Young, as well as Emily Esfahani Smith and Rhonda Magee.

Central to our study is the inquiry into the enduring relevance of foundational texts from the first millennium in today’s contemporary landscape and how these might offer cognitive insights into modern spiritual practices, and the search for meaning. Despite the marked differences in historical and cultural contexts between these early texts and the present day, they contain timeless themes that continue to strike a chord with individuals seeking, spiritual growth, purpose and insight. Prominent figures engaged in the interpretation and application of these texts include the esteemed spiritual teacher, Andrew Harvey, as well as three distinguished faculty members from the Center for Action and Contemplation in Albuquerque, Cynthia Bourgeault, James Finley and Richard Rohr. Notable voices in this realm also encompass luminaries such as Karen Armstrong, Mirabai Starr, Wayne Teasdale, and the revered Trappist monk and scholar, Thomas Merton. It is around the profound contributions of Merton that our study pivots; serving as the formative focal point of our study.

### 3.2 Background on Merton

Thomas Merton was a writer, theologian, mystic, poet, social activist and scholar of comparative religion. Born in January 1915, he became a Trappist monk after a radical conversion experience, where-after he was ordained to the Catholic priesthood in May 1949. Until his death in December 1968, Merton was a member of the Abbey of Our Lady of Gethsemani, near Bardstown, Kentucky.

Over 27 years Merton wrote more than 50 books on spirituality, social justice and a quiet pacifism. Among his most enduring works is his autobiography *The Seven Storey Mountain* (Merton, 1948). In time Merton became a keen proponent of interfaith understanding, exploring Eastern religions and comparing these to Christian mystical practice; subsequently pioneering dialogue with prominent Asian spiritual figures, including the Dalai Lama; Japanese writer D.T. Suzuki; Thai Buddhist monk Buddhadasa, and Vietnamese monk Thich Nhat Hanh. From there he went on to write books on Eastern faith-sets and how Christianity related to them; this at a time when attempts at such integration were highly unusual, especially within established religious orders. In his biography on Merton, the theology-scholar, Cunningham (1999), cited him as perhaps the most important spiritual writer-and-master of the last century.

### 3.3 Recording background

During the latter part of his life, Merton undertook the training of noviciate monks. A recording of his teaching sessions was released in 2012, capturing a series of his lessons delivered over the period 1962 to 1964 (Merton, 2012). The lesson critical to this study was delivered on 19 May 1963 on the topic “Cassian on Prayer.” Separated by 15 centuries, John Cassian (AD 360–435) was a Christian ascetic acknowledged for his mystical writings and his monastic practices. Cassian was one of the so-called “Desert Mystics,” who, after the mainstream formalization of Christianity by Constantine in 313, withdrew into the arid areas of Egypt, Syria, Palestine and eastern Turkey to practice a pared down version of their faith. Key to this practice was a psychologically contingent version of centering prayer and a contemplative lifestyle (Rohr, 2015). Raised in Asia Minor as part of this desert tradition, in the last two decades of his life, Cassian founded a monastery in vicinity of Marseilles, in southern France. There, in about 420, he wrote a piece dealing with spiritual training entitled: *The Conferences of the Desert Fathers*. Comprised of 24 section-conferences, and originally in Latin, *The Conferences* were subsequently translated into Greek, and rapidly integrated within the traditions of Eastern monasticism (Harmless, 2004). Conferences 9 and 10, on prayer, especially “fiery prayer,” is the focus of Merton’s teaching in the May 1963 recording under study.

### 3.4 Forms of prayer

In this lesson explaining to his noviciates the contemplative and mystical prayer-forms, Merton’s rendering of Cassian’s text moved beyond just words to focus especially on meaning, context and interpretation, so as to convey a richer, more accurate rendering of the original Latin into English. So doing, in the recording Merton observed that prayer, in any form, is inherently intuitive and does not consist of reasoning (audio timing: 1:12). True prayer, he stated, is about “letting go” (1:13), about turning back to God, “like an apple thrown back into a barrel from whence it came” (1:15). Prayer may be considered as a form of abandonment, annihilation even, where “you simply fall apart and collapse into God” (1:16). Merton offered too that it may be considered as communication with one’s own father, one’s own personal truth (1:17).

#### 3.4.1 Ordinary contemplative prayer (little-p)

After outlining these fundamental attributes of prayer, Merton went on to draw a clear distinction between the lower forms of contemplative prayer and the loftier realm of mystical prayer, here denoted as little-p and Big-P, respectively. He characterized little-p as a state of being, a form of everyday prayer that is readily accessible to all individuals (1:19). This baseline format inherently embodies a passive and pre-mystical nature (1:20). Within the realm of contemplative prayer, Merton identified four distinct states, forming a progressive hierarchy. At the base, we find the prayer for pardon (*obsecratio*) (1:24), aimed at achieving absolution from guilt. Once this state is reached, it paves the way for the second state, the prayer for spiritual needs (*orationes*), attainable through a level of spiritual confidence and fluency. Subsequently, the third state emerges—the prayer for others (*postulationes*)—achieved through a profound commitment to the well-being of others. Finally, at the apex of the contemplative prayer states, we find the prayer of thanksgiving (*gratiarum actio*) (1:25).

#### 3.4.2 Higher mystical prayer (Big-P)

Having thus described the sequential little-p prayer hierarchy, Merton elaborated on how its hierarchy may be short-circuited by an intervening moment of higher mystical Big-P prayer:

but here’s the thing that is interesting – what people have not noticed so far in Cassian. … Here’s something he says that is not like all the others – and this I think is very, very good – you have got these four states – four kinds of prayer—but he says it can happen that in any one of these four states you can suddenly get hit – so to speak – by lightning – and you go zing! And you go out of the picture and you are on top of everything—you can go from the lowest kind of prayer into this—or from the second kind—or from the third kind—or the top kind—and if you are hit by this—at any level of the spiritual life—you go beyond all levels—but you get into a wholly different sphere in which all graces of all levels are fulfilled at once (1:26–1:27).

With reference to the quotation above, for the widely read Merton, it seems that this feature of Big-P prayer had remained unrecognized until his discovery of it, and our review of pre-existing and subsequently published English examinations of the Cassian text appears to corroborate Merton’s claim of novelty.^1^

Introduced as such, Merton then characterised mystical prayer as not a state, as per the earlier versions of contemplative prayer, but rather an act (1:27). In duration it may last thirty seconds to two minutes or even five minutes, but no longer (1:27). In such moments, earlier states of little-p become irrelevant. With no little-p hierarchy, to quote Merton, you are “in no-man’s land” (1:28). Translating Cassian from the Latin, Merton states:

This is something quite original: “*Yet sometimes the mind which is advancing to the true state of purity, and has begun to be rooted in it*”—see—always this idea of purity of heart in the background—“*can conceive all these kind of prayers in a single action. It cannot be understood but may be compared to the leaping of a flame. It consists of a powerful and wordless pouring forth of prayer to God, which the Spirit, with groanings that cannot be uttered, sends up, though not conscious of its content. In that moment it conceives and puts forth what no one can describe, and which the mind*—*apart from that moment*—*cannot remember. So it happens that whatever state of life a man has reached, he sometimes can offer pure and devout prayer*” (italics: quoting Cassian directly, 1:29).

Explaining the Cassian text above, Merton continued: “When he’s talking about pure prayer, he’s talking about this act in which one is grabbed by God and taken out of his state and put into another condition and put back again. Translating Cassian’s Latin, again he continued: “*So it happens that whatever state of life a man has reached: he sometimes can offer pure and devout prayer; even the lowliest place where a man is repenting from fear of punishment.”* Qualifying Cassian, he stated: “see—even the degree of fear—where man is simply a slave—he can suddenly be wrapped up in this” (1:29).

At this point Merton expanded on the nature of this mystical prayer, noting that it is a free gift from God, “when God gives what he wants to whom he wants” (1:30). Critically he noted that the higher points of the ordinary prayer hierarchy are not important here: “This also means that it is not important, really, to get yourself up into a high degree of prayer. The thing is to pray with your whole heart and with all sincerity on the level where you are. That’s all that’s important. So that I think is very beautiful” (1:30).

Merton explained further that mystical prayer is momentary and transient (1:31), seldom understood (1:33), or remembered, and that it is explained, if at all, by a condition of humility and a shedding of ego and self-consciousness (1:35). He concluded this section of the lesson by quoting and explaining St. Anthony’s (251–356) view that prayers of this form cannot be owned or shaped or mastered:

“*That prayer is not perfect in which the monk understands himself and the words that he is praying*” – you see what St Anthony is saying … is that in this pure prayer it is not a question of being exceptionally aware of yourself. … Therefore if you think it’s something you can take away – see – and you sort of hug it to your heart – and take it off to a little corner some place – and take it out and look at—see – this is mine—see – that is not the kind of thing it is at all. … There’s a radical difference between this act of prayer and any other time – and the difference is so radical that you cannot even remember a lot of it (italics: quoting Anthony directly, 1:36).

### 3.5 Extrapolating the desert mystic narrative

What becomes apparent from analysing the Merton transcript is that Big-P prayer is a rare phenomenon, transient in nature; not earned, not owned, not understood, and not remembered. While considered the highest version of prayer, in the practice of prayer one does *not* set out to engage in mystical prayer. The common goal is rather prayer, pure and simple, and here versions of little-p typically prevail.

To be clear: you can have all the lower levels of little-p and still never activate an instance of Big-P. All you can do is prepare yourself, to “set the table” as it were. Once set, something different might happen, but this is irrespective of one’s efforts or spiritual evolvement. To rephrase: “setting the table” is a metaphor equivalent to the laying of groundwork, or the priming of a canvas; describing a requisite degree of inner preparation, of locating one’s inner authentic self. It is the gateway to Big-P, though it does *not* guarantee entry. Common requirements to entry are: authenticity, getting your mind out of the way, whole heartedness, sincerity, and the absence of ego. Rohr (2023) speaks also of having the mind of the child: a *tabula rasa*—a willingness to be in constant awe and excitement. For Rohr (2023), this is the place where “someone who’s not in their mind yet”; a place of nakedness, alertness and surrender, a characterization which ties with the Zen concept of “*shoshin*,” or beginner’s mind, where there is no need for attainment.

Suzuki (2020), a contemporary of Merton, emphasizes that if the mind becomes overly demanding or greedy, it loses its depth and self-sufficiency. Here, for Rohr, the essence lies not in earning or achieving, but in cultivating relationships over results or stringent requirements. As Rohr (2003, 33) succinctly states, “Once we perceive, the rest naturally follows.”

**Figure 1:**
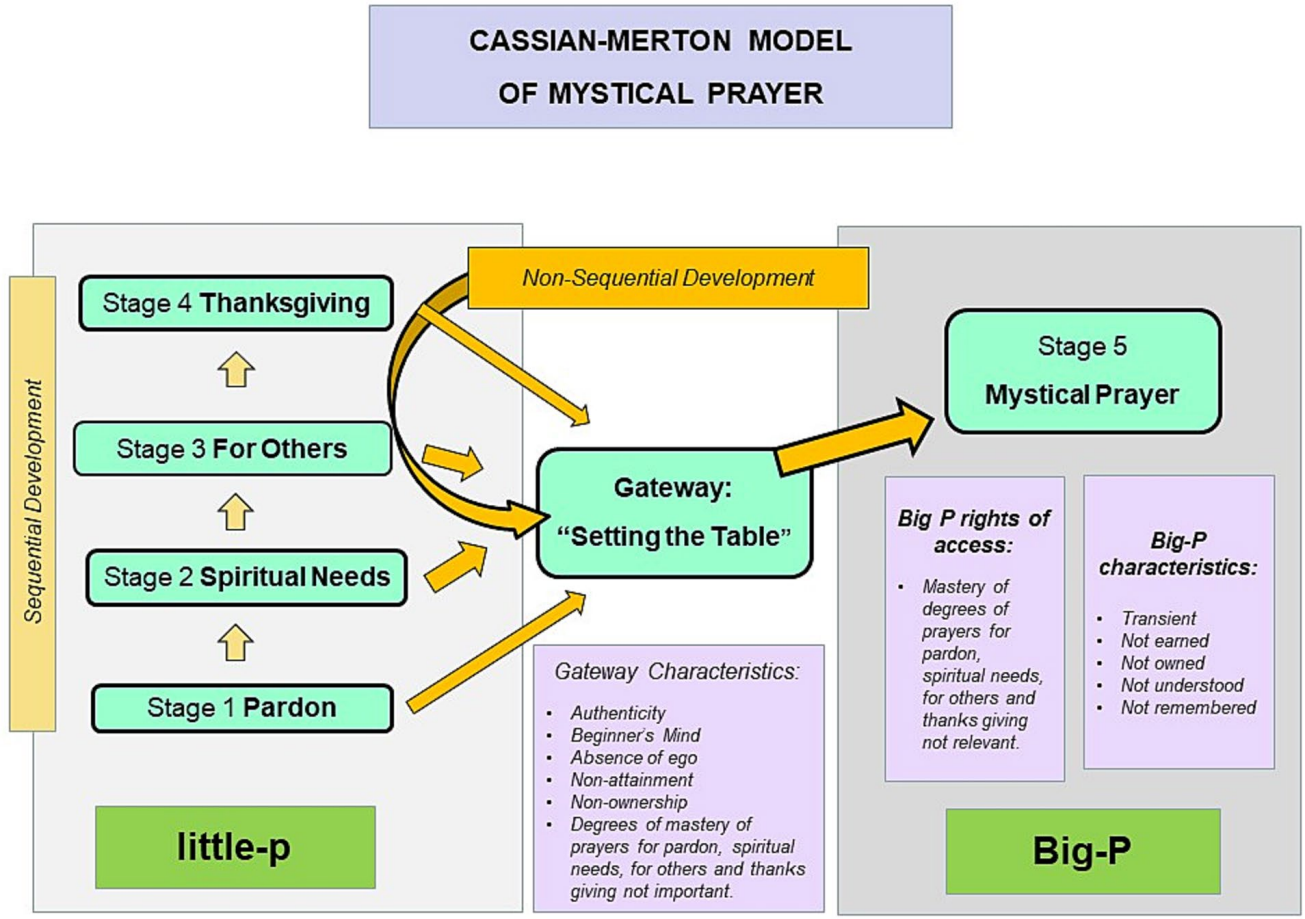
The Cassian-Merton model of mystical prayer. Note that stage 5 is not attained as a progression but received as a gift.

Figure 1 depicts the prayer dynamic outlined above.

## 4 Comparison with the musical domain

### 4.1 The little-C/Big-C format in music

Relative to the little-p/Big-P of mystical religion, in contemporary art, the little-c/Big-C continuum is better understood and more widely applied, especially in the creation of music. Within song-writing, the process of creation typically begins with little-c—where the amateur composer is merely trying to find some kind—any kind—of expression. With skilled writing professionals, given sufficient perseverance and practice, little-c outputs may even morph into Pro-c outputs. Big-C moments are comparatively harder to conjure, and may involve additional non-creative inputs such as reputation and luck (Runco, 2014). That said, while Big-C creativity is usually followed by some broader market recognition—this may not necessarily hold. The 1970 Sixto Rodriguez album *Cold Fact*, is such a case. Though it hardly registered in the US, unbeknown to Rodriguez, in Australia and Apartheid South Africa, the album found broad acceptance as a remarkable piece of creative work. In 2012 it attained global recognition in the Oscar winning documentary, *Searching for Sugar Man*. Generally speaking, instances of Big-C creativity may happen to anyone, whether they are famous or not. As per Runco (2014, p. 132), outputs from Big-C and lesser versions of creativity “differ mostly in things that occur after the act”.

Cognizant of these considerations, in this study we limit our data to instances of *subjectively* reported Big-C outputs by recognised artists (Stein, 1953). Though not universal in scope it does offer a sample of undisputed greatness. What now becomes important *is how we explain the developmental path of creativity and whether and how Big-C creativity differs from those at the lower rungs of such development.*

### 4.2 Models explaining little-C creativity

Early theories on the development of creativity are typically presented as a set of sequential steps. The earliest model by Wallas (1926) proposed a four-stage model negotiating stages of preparation, incubation, illumination, and verification. The Torrance model (Torrance, 1988, 1995) also included four stages: defining the problem, hypothesis formation and then testing and finally communication of output; while Amabile’s (1983) model posited a five-stages involving problem presentation, preparation, response generation, response validation and outcome. The Treffinger Creative Problem Solving six-stage model (Treffinger, 1995) spanned mess finding, fact finding, problem finding, idea finding, solution finding, and finally acceptance finding.

While these sequential models are helpful in explaining the process of idea creation, the addition of biographically derived antecedents of creativity also contribute to explain the process that leads to meaningful creative output. Amabile’s (1983) model, for example, assumed that various factors like intrinsic motivation, domain knowledge and skill exerted their effects on different stages of the process. Likewise, Simonton’s model (Simonton, 2014) argued that creative performance is determined by deliberate practice and driven primarily by dispositional traits and cognitive abilities, as well as genetic and environmental factors. The resolution of creative performance, concluded Simonton, is certainly non-sequential and probably best described through the above factors via a structural equation model-styled analysis.

Supplementary to Amabile (1983) and Simonton’s (2014) incorporation of biographical markers, are the so-called evolutionary models of Campbell (1960), Simonton (1999), and Priilaid and Callaghan (2023). These introduce Darwinian ideas of creativity as a recursive process of random idea variation and the selective retention of those that seem most likely to succeed.

Drawing additional insights from Gardner’s (2011) theory of creative asynchrony, Priilaid (2023) and Priilaid and Callaghan (2023) describe how formative stages of creative development (namely Pathology, The Rage to Master, and Authenticity) carry with them important dispositional antecedents and personality attributes. Thus on one hand, creatives may be overly susceptible to anxiety and psycho-pathology while on the other, they may also exhibit notable levels of self-confidence, independence of judgement and commitment (Feist, 1998, 2010, 2019). These early emotional markers, argue Priilaid and Callaghan (2023), serve as the primal drivers of key creative behaviors including perseverance (see also Forgeard, 2024), practice, the development of fluency and insight, and possibly flow.

At this point it is important to grasp that here the creative output is always tentative, and almost always limited to little-c. In this liminal space no one is *actively* seeking to write a Big-C song. Everyone is just emerging; always with a goal just to express as best as one may. As an exemplative anecdote, the great Eagles song-writer, Glen Frey, relates the story of how, when aged 19 in Detroit, his 21-year-old mentor at the time, Bob Segar, advised him to simply keep writing. On interview (Ellwood, 2013), Frey recalled how Segar had said to him: “You know, if you want to make it you have to write your own songs. And I said, well, what if they are bad? And he said, well they are going to be bad; so just keep writing and eventually you’ll write a good song.” As per this insight, it is clear that song writing is a stumbling process of just writing regularly. Reflecting on his craft, Frey (2015) said: “I do not think you should work thinking about hit songs. The only thing you have to do is you have to write. And you have to write every day. So when I do write I make sure I do it every day.”

To summarise to this point, the developmental pathway to little-c versions of creativity may be fairly described as approximately path dependent; initiated by some form of emotional spur; from which would follow habits of perseverance and practice; out of which would flow a degree of expertise from which perspective, voice and professional mastery would ultimately emerge. Given this developmental trajectory, how then does one transit from regular moments of little-c, to less-common instances of the higher version of Big-C? To paraphrase Lubart (2001), who asked how process-related differences may lead to different levels of creative performance, the vexed issue here is what the preconditions of Big-C might be, and how a conversion from such preconditions to Big-C might be triggered.

### 4.3 Higher (mystical) creativity (Big-C)

From the outset we stipulate that Big-C is dichotomously distinct from lesser forms of creativity. As Neil Young puts it: “It’s not a craft. Crafts usually involve a bit of training and expertise and you draw on your experiences” (McDonough, 2002a,b, p. 127). Young’s inference is that training, expertise and experience do not guarantee access to the Big-C and there is simply no straight forward path from regular craft-type mastery to outputs of Big-C. It is no surprise therefore that irrespective of perseverance and practice, domain-specific experts struggle to transit automatically to upward moments of Big-C (Simonton, 2000). In the literature such moments might be equated to “flow”. Nakamura and Csikszentmihalyi (2002, p. 89) reference the flow concept, with its origin in the work of Csíkszentmihályi in the 1960s:

Csíkszentmihályi was struck by the fact that when work on a painting was going well, the artist persisted single-mindedly, disregarding hunger, fatigue, and discomfort-yet rapidly lost interest in the artistic creation once it had been completed. Flow research and theory had their origin in a desire to understand this phenomenon of intrinsically motivated or autotelic, activity rewarding in and of itself (auto = self, telos = goal), quite apart from its end product or any extrinsic good that might result from the activity.

While quite how flow is accessed remains one of the great mysteries, in conditions of flow, the subject is likely to experience intense and profound concentration, a merging of action and awareness, and an absence of reflective self-consciousness and control where creativity “seamlessly unfolds from moment to moment” (Nakamura and Csikszentmihalyi, 2002, p. 90). In their model of creative development Priilaid and Callaghan (2023) suggest that as a foregrounding, though not a guarantee for accessing flow, the artist should proverbially “set the table,” a process which requires a degree of inner work, reorientation, and preparation. Neil Young described this preliminary process well: “And sometimes I cannot get them (songs) to come, y’know, but then if I just get high or something, and if I just sit there and wait, all of a sudden it comes gushing out. I just got to get to the right level. It’s like having a mental orgasm” (McDonough, 2002a,b, p. 126). By contrast, Leonard Cohen has struggled to cross the Big-C threshold: ‘I never had the sense that I was standing in front of a buffet table with a multitude of choices…I felt I was operating in more what Yeats used to say was the “foul rag and bone shop of the heart.” I just pick it together. I do not work with a sense of great abundance’ (Cocker, 2012). Donald Fagen, from Steely Dan, speaks also of his frustration at the problems of repeatedly accessing moments of flow over time: “That’s what most artists have, just a couple of years. You’re very lucky if you have them, and very lucky if you can maintain such standards. There are only a few people, great artists, who work until they die. Stravinsky or Vladimir Nabokov” (Sweet, 1994, p. 198).

With little definitive literature on how the Big-C may be accessed, as our base-data we deploy song-writing accounts of acknowledged singer-song-writers from the 60’s, 70s and 80’s. From these, three features emerge as factors that characterise the Big-C. *Firstly*, Big-C songs emerge with little or no preamble. *Secondly*, the process appears to be unconscious, with minimal rational intervention. There is a dream-like quality to their emergence, and most major artists appear to have accessed their dream-world in the writing of some of their best music. *Thirdly*, it seems that the Big-C music exists beyond them in some universal bank or repository. To this extent musicians all attest to an almost total sense of non-ownership of their great songs. Further extrapolated below, each of these factors point to one crucial governing condition of the Big-C: which is a total lack of agency, or control, or ego on how high-level creativity is accessed.

#### 4.3.1 Big-C emerges with no preamble

The first key feature of Big-C is that it emerges as a state of spontaneous and inspired non-conscious action. As per Table 1 below, the jazz pianist Bill Evans eloquently relates Japanese calligraphy to the spontaneity of jazz: ‘these artists must practice a particular discipline, that of allowing the idea to express itself in communication with their hands in such a direct way that deliberation cannot interfere.’ Likewise, the popular song writer Barry Gibb likens song composition to a flash of an idea, or a flash of a chorus of a song, which can come literally in the middle of the night. Getting into such a ‘flash space’ can be key to unlocking the creative experience. His brother Maurice describes getting into a zone with the others, a creative chain, which if broken was problematic. Most of their successful songs were written quickly. When recording, Springsteen would avoid over preparation and to just go with the flow: Said he: ‘It’s fascinating to record a song when musicians do not know it…if people learn their parts too well, they consciously perform rather than play flat out.’ Paul Simon reports that his “Bridge over Troubled Water” came out so fast that he did not know where it came from, while Graham Nash reports how his recording of Jerry Garcia’s pedal steel guitar on “Teach Your Children” was done in one miraculous take.

**Table 1:** Big-C artists explain the absence of preamble.

1 Bob Dylan: *Setting the table by avoiding preamble, reveals a flash-space*: Following a freak motorbike accident in July 1966, Dylan sought sanctuary on a farm close to Woodstock. Dismissive of the popular psychedelic format, songs from the 1968 album, *John Wesley Harding*, were pared-down and countrified, taking took only nine hours to record. Dylan’s drummer, Kenney Buttery, confirmed the new inclination towards the less polished format: “We went in and knocked ‘em out like demos. It seemed to be the rougher the better. He would hear a mistake and laugh a little bit to himself as if [to say], ‘Great, man, that’s just great. Just what I’m looking for.’” (Sounes, 2011)
2 Bruce Springsteen: *Setting the table (do not over-learn) reveals a flash-space:* Springsteen is similar in respect to his recording sessions: no over preparation, just going with the flow and staying with the vibe. Said Springsteen on the subject: “It’s fascinating to record a song when musicians do not know it. … if people learn their parts too well, they consciously perform rather than play flat out. When you just launch into it, it breaks down another barrier between you and the audience. One less layer of formality.” In a recent studio session, Springsteen was seen shushing the band to silence and gently admonishing them: “That’s good! If it gets any better than that, it’ll be worse.” (Carlin, 2012)
3 Paul Simon: *Setting the table (sitting in a darkened bathroom) reveals a flash-space*: “Bridge over Troubled Water” came very quickly to Paul Simon. So fast did he compose it that he asked subsequently: “Where did that come from? It does not sound like me.” (Eliot, 2010, p. 105)
4 Barry Gibb (Bee Gees): *Setting the table (at night) reveals a flash-space*: “What songwriting’s always been to me is basically like a flash. I have a flash of an idea or a flash of a chorus, or a flash of a song before it’s actually constructed. That has not changed, it’s continued right through my life. I’ll get up in the middle of the night and put something on a dictaphone and go back to sleep.” (Bee Gees, n.d.)
5 Ray Davies on Jimmy Webb: *Setting the table (by living in a car) reveals a flash-space*: asked whether the muse had stayed with him over time, Ray Davies of The Kinks carefully replied: “To a degree. … Alone in a quiet room I tend to be too reverential of the space needed. It’s the old Jimmy Webb theory: apparently when he wrote “Up, Up and Away” and the hits for the *5th Dimension*, he lived in a car and had a very transient lifestyle. According to folklore he had all the success, bought a fantastic house, put a studio in it and then could not write.”(Rachel, 2013, p. 8)
6 Graham Nash: Setting *the table (by avoiding preamble,) reveals a flash-space*: talking about “Teach Your Children” off Crosby et al. (1970): The song features a pedal steel guitar solo from Jerry Garcia. According to Wally Heider, while CSN&Y were in one studio, the Jefferson Airplane were in another studio, and the Dead were in another studio. “And so we were all hanging out. We got to “Teach Your Children” and we put our voices on it and Stephen (Stills) goes, “You know, a guitar solo does not feel right for this.” Crosby said, “Hey, I heard Garcia is playing pedal steel. Why do not you ask him to try a pedal steel solo? … So he brought his pedal steel in, he set it up, we recorded the first track and I said, “That was fucking great, fantastic.” He said, “You know, I fucked up a little in the chorus. Can we do a second take?” I said, “Absolutely, we can do a second take. But I’ll tell ya, I’m not going to use it.” (Runtagh, 2018)
7 Maurice Gibb (Bee Gees): *Setting the table (at night in a collective) reveals a flash-space*: “It’s something that we have been gifted with, and we have just nurtured all the years. It’s something that happens, and we all get into the same ‘zone.’ … That’s why if one of us is a little off that night, it’s like, ‘I do not feel too good tonight.’ We do not write. Because we need that input. We need to be joined like a chain. If there’s a broken link, we cannot do it right.” … “Most of the songs we have written that have been successful have been written quickly.” (Bee Gees, n.d.)
8 Lady Gaga: *Setting the table (dropping the needle) reveals a flash-space*: the rapidly aging and infirm saxophonist Clarence Clemons, for years one of Bruce Springsteen’s sidesmen was asked to play some sessions with Lady Gaga. When he asked her what and how she wanted him to play, she replied “‘Just be Clarence Clemons. Play what you want, be who you are. I’m gonna drop the needle and you go.’” Smiling, he recalled: “So that’s what I did and she loved it. That was very cool. Something I had not experienced in a long time, not since Bruce’s first albums. Sit down and play, just play. It reminded me of why I love being a musician and doing what I do.” (Carlin, 2012, p. 444)
9 Bill Evans: His linear notes to his 1959 album *Kind of Blue* illustrate this by describing a style of Japanese calligraphy that requires brushwork using watered-down black paint over a thin tissue-like parchment. Erasure is impossible and the visual integrity of the work is destroyed if the brushstroke is hesitant, unsure, overly deliberate or unnatural. Just as easily, a lack of skill can rupture the delicate parchment. Because of these constrictions, wrote Evans, “these artists must practice a particular discipline, that of allowing the idea to express itself in communication with their hands in such a direct way that deliberation cannot interfere.” (Evans, 1959)

#### 4.3.2 Big-C process appears to be unconscious and irrational

A careful study of the biographical evidence suggests also that there is a well-recognised state of unconscious or sub-conscious receptivity that plays a key role in achieving Big-C, with musicians reporting the attainment of a state of unthinking creativity. As per Table 2, a case in point is Neil Young, who, with a bad dose of flu, and in an almost delirious semi-conscious state, in a single day wrote ‘Cinnamon Girl’, ‘Cowgirl in the Sand’, and ‘Down by the River’. According to Young, ‘I do not feel the need to write a song. It’s not like that. It’s almost like the song feels the need for me to write it and I’m just there. It’s not like I’m doing a job. Songwriting, for me, is like a release. … If you are thinking … while you are writing, do not! If I can do it without thinking about it I’m doing great’ Likewise, Bob Dylan, explains that: ‘If your mind is intellectually in the way, it will stop you.’ In the words of Keith Richards: ‘With songwriting it’s a constant experiment. I’ve never done it consciously, like saying, I’ve got to explore such and such a thing.’

**Table 2:** Big-C artists explain the role of the unconscious.

1 Bob Dylan: “If your mind is intellectually in the way, it will stop you,” said Dylan. And so you have to train your brain to let go and not to theorise too much. Looking back at the time when writing came easily, Dylan reflected on the process of songwriting: “Still staying in the unconscious frame of mind, you can pull yourself out and throw up two rhymes first and work it back. You get the rhymes first and work it back to see if you can make sense of it another way. You can still stay in the unconscious frame of mind to pull it off, which is the state of mind you have to be in anyway.” (Ricks, 2003)
2 Neil Young: “I do not feel the need to write a song. It’s not like that. It’s almost like the song feels the need for me to write it and I’m just there. It’s not like I’m doing a job. Songwriting, for me, is like a release. It’s not a craft. Crafts usually involve a bit of training and expertise and you draw on your experiences – but if you are thinking about that while you are writing, do not! If I can do it without thinking about it I’m doing great.” (McDonough, 2002a,b)
3 Keith Richards: “With songwriting, it’s a constant experiment. I’ve never done it consciously, like saying, I’ve got to explore such and such a thing.” (Richards, 2010)
4 Bill Evans: Evans noted that learning jazz required relocating the technical problems of playing from the outer layer of cognition to the inner level of the unconscious. “Now when that becomes subconscious then you can begin concentrating on that next problem which will allow you to do a little bit more – and so on and so on. … We must remember that in an absolute sense jazz is more of a certain process of spontaneity than a style,” he said (Cavrell, 1966)
5 Tim Rice-Oxley (Keane): *Setting the table (in the country) reveals flash-space*: talking to his song “Somewhere only we know,” Keane pianist/song-writer Tim Rice-Oxley, said: “The song is about us being back and having something to cling to. I picture a particular place in Sussex, just a bit of scrub where we used to go when we were kids. There was a fallen pine tree and it seemed like a place to escape from the reality of the band’s failure that seemed to be fast approaching. Richard [Hughes, drummer] recently sent me a photo of the three of us on that exact spot, when we were 11 or something, and I wonder if I’d subconsciously remembered the photo when I wrote the song.” (Simpson, 2019a)
6 Mick Hucknall (Simply Red): *Writing good music in a stream of consciousness*. As a teenager, in 1978, Hucknall wrote the 1985 hit, “Holding Back the Years,” which subsequently reached number 1 on the Billboard Hot 100. Taking up the story, Hucknall says “At art school, a teacher said: “The best paintings are when you get lost in a piece of work and start painting in a stream of consciousness.” I wanted to do music, not art, so started writing lyrics that way. The first song I wrote was called Ice Cream and Wafers. The next was Holding Back the Years. I did not realise what it was about until I’d finished it.” (Simpson, 2018)

As an adjunct to this semi-conscious component of Big-C, significant musician-artists also report a dream-like state when composing some of their material (see Table 3). Richards recorded the chord structure for the Rolling Stones hit ‘Satisfaction’ while experimenting late at night before falling asleep, leaving the recorder on. Similarly, he says, “‘Wild Horses’ almost wrote itself…It’s like ‘Satisfaction.’ You just dream it, and suddenly it’s all in your hands.” According to Paul McCartney, the melody for ‘Yesterday’ was composed in a dream, and so it was for John Lennon with the song ‘#9 Dream’, for Robin Gibb with ‘You Win Again’, and for Jimi Hendrix for ‘Purple Haze.

**Table 3:** Big-C artists explain the influence of dreams.

1 Bob Dylan: *Writing good music from a dream.* Dylan wrote “One more cup of coffee” off Desire (1976) from a dream (Scorsese, 2019).
2 Neil Young: *Writing good music while delirious.* On one day in 1968, during a bad dose of flu, a half delirious, semi-conscious Neil Young wrote three epic tunes: “Cinnamon Girl”, “Cowgirl in the Sand” and “Down by the River” (Young, 2013).
3 Graham Nash: *Writing good music while delirious.* “There’s a big difference between smoking dope and drinking beer — a huge difference. I was smoking dope and the Hollies were all drinking beer. I was getting high at the Oulton Motel after this particular show in Leeds [in 1968], and I just wrote “Lady of the Island,” “Right Between the Eyes” and part of “Teach Your Children.” It happened all in one night (Runtagh, 2018).
4 Keith Richards: *Writing good music is dream-like. “*“Wild Horses” almost wrote itself. … It’s like “Satisfaction.” You just dream it, and suddenly it’s all in your hands.” (Richards, 2010)
5 John Lennon: *Writing good music from a dream.* The song, “#9 Dream” came to Lennon in a dream. Said Lennon to the BBC in 1980: “That’s what I call craftsmanship writing, meaning, you know, I just churned that out. I’m not putting it down, it’s just what it is, but I just sat down and wrote it, you know, with no real inspiration, based on a dream I’d had.” Said May Pang, girlfriend of Lennon, “This was one of John’s favorite songs, because it literally came to him in a dream. He woke up and wrote down those words along with the melody. He had no idea what it meant, but he thought it sounded beautiful. John arranged the strings in such a way that the song really does sound like a dream. It was the last song written for the album, and went thru a couple of title changes: So Long Ago, and Walls & Bridges.” (Beatles Bible, 2010)
6 Paul McCartney: *Writing good music from a dream.* The melody for “Yesterday” was composed in a dream. His first worry was that it wasn’t original. “For about a month I went round to people in the music business and asked them whether they had ever heard it before. Eventually it became like handing something in to the police. I thought if no one claimed it after a few weeks then I could have it” (Cross, 2005). Until the lyrics were fully composed, McCartney substituted “Scrambled Eggs” into the words. The opening verse ran: “Scrambled eggs/Oh my baby how I love your legs/Not as much as I love scrambled eggs.” (Miles, 1997)
7 Robin Gibb (Bee Gees): *Writing good music from a dream.* Robin Gibb developed the melody for their 1987 hit “You Win Again” when he awoke from a dream with the song’s key line and melody running through his head. He was able to locate a tape recorder and so sang the line into the machine: “There’s no fight you cannot fight/This battle of love with me/You win again” (Rachel, 2013, p. 25). When asked when he preferred to write his music, replied Gibb: “I would have to say the early hours of the morning when it’s quiet, about 2 a.m. You cannot write when there are people around. It’ a vexation to the spirit” (Rachel, 2013, p. 26)
8 Jimi Hendrix: *Writing good music from a dream.* After reading a science fiction novel, the words to “Purple Haze” (1967) came to Hendrix in a dream (Wegman, 2000).
9 Lloyd Cole and the Commotions: *Writing good music from a dream.* On describing how “Rattlesnakes” (1984) was written, guitarist, Neil Clark, recalled: “I woke up one morning with the riff for Rattlesnakes in my head. I said to my girlfriend: “I’ve got to get this down on the Portastudio NOW!” She was like: “You’ve what?” (Simpson, 2019b)

#### 4.3.3 Big-C music is externally vested in a universal repository

Finally, as show-cased in Table 4, artists identified in this study report that their truly great material is accessed from a place beyond themselves. According to Dylan, ‘The songs are there. They exist all by themselves just waiting for someone to write them down. I just put them down on paper. If I did not do it, someone else would.’ According to him, the songs ‘just come through me…It wasn’t like I was having to compose them’. Asked whether he believes music is a product of the mind alone, Pink Floyd’s David Gilmour responded along the same lines: “I’m an atheist, so I hate to say it out loud, but there are times when it feels like music is channelling itself when I’m writing. It does not always feel like it’s something I’ve done – it’s somehow just comes through me” (Lord, 2024). Similarly, said Keith Richards: ‘…And it’s that weird mixture of your actual rock and roll and at the same time this weird echo of very, very ancient music that you do not even know. It’s much older than I am, and that’s unbelievable! It’s like a recall of something and I do not know where it came from’. As Bowie explains, ‘Searching for music is like searching for God. They’re very similar. There’s an effort to reclaim the unmentionable, the unsayable, the unseeable, the unspeakable, all those things, comes into being a composer and to writing music and to searching for notes and pieces of musical information that do not exist’. According to Amabile (2011, p. 3), creativity is “the production of a novel and appropriate response, product, or solution to an open-ended task” which is “appropriate to the task to be completed or the problem to be solved; that is, it must be valuable, correct, feasible, or somehow fitting to a particular goal.” The problem to be solved by our subjects is however seemingly mystical, or requires a mystical perspective to be open to the necessary inspiration. For Leonard Cohen, ‘We all know that the activity depends not just on perseverance, perspiration but also a certain kind of grace and illumination.” It is precisely this mystery of where the source of creativity is that makes the study of this source so important. Paraphrasing further from Table 4, James Taylor admitted that ‘it’s such a mysterious and sub-conscious process that I could not really say that I wrote those songs. I just channelled them.” For Paul Simon the process was similar: ‘…there are times when … you are plugged into the universe and all of a sudden something comes through you, and it’s yours but it is not yours.”

**Table 4:** Big-C artists explain the role of mystery and external agency.

1 Bob Dylan: “The songs are there. They exist all by themselves just waiting for someone to write them down. I just put them down on paper. If I did not do it, someone else would” (Turner, 1962). He is reported to have told Steve Jobs about the songs he wrote back in the 60s and 70s. “They just came through me,” he told Jobs. “It wasn’t like I was having to compose them” (Isaacson, 2011).
2 Keith Richards: “But Flash is particularly interesting. “It’s all right now.” It’s almost Arabic or very old, archaic, classical, the chord setups you could only hear in Gregorian chants or something like that. And it’s that weird mixture of your actual rock and roll and at the same time this weird echo of very, very ancient music that you do not even know. It’s much older than I am, and that’s unbelievable! It’s like a recall of something, and I do not know where it came from” (Richards, 2010, p. 210)
3 Bill Evans: “I believe that all people are in possession of what I call a universal musical mind. Any true music speaks with this universal mind to the universal mind in all people. The understanding that results will vary only insofar as people have or have not been conditioned to the various styles of music in which the universal mind speaks. Consequently, some effort and exposure is often necessary to understand some of the music from a different period or a different culture – the knack to which the listener has been conditioned” (Cavrell, 1966).
4 Leonard Cohen: “When I was packing in Los Angeles, I had a sense of unease because I’ve always felt some ambiguity about an award for poetry. Poetry comes from a place that no one commands, that no one conquers. So I feel somewhat like a charlatan to accept an award for an activity which I do not command. In other words, if I knew where the good songs came from I would go there more often” (Cohen, 2011) “We all know that the activity depends not just on perseverance and perspiration but also a certain kind of grace and illumination…” (Cocker, 2012)
5 David Bowie: *Searching for God.* “Searching for music is like searching for God. They’re very similar. There’s an effort to reclaim the unmentionable, the unsayable, the unseeable, the unspeakable, all those things, comes into being a composer and to writing music and to searching for notes and pieces of musical information that do not exist” (Macatee, 2016)
6 Elvis Costello: *Writing good music is like being a lightning rod.* “I do not want to sound spiritual, but I try to make an antenna out of myself, a lightning rod out of myself, so that whatever is out there can come in” (Montandon, 2007)
7 James Taylor: *Writing good music is like channelling.* Speaking about the truly great songs he’s written like “Sweet Baby James,” “Fire and Rain” and “Carolina On My Mind,” Taylor confesses: “Yes they are good songs, I still sing them, I still connect with them, and I am thrilled to have written them. But I do not really feel as though I write songs, I feel as if I hear them first, and remember them and get them down. But it’s such a mysterious and sub-conscious process that I could not really say that I wrote those songs. I just channelled them; they happened to me first. … There is a sort of lightning bolt kind of moment when you are visited by a song; sometimes a whole song, sometimes just a fragment. And you have to collect those fragments and later on sequester yourself, hideaway somewhere and work them.” (Taylor, 2011)
8 Barry Gibb (Bee Gees): *A vault of instinctive music.* “There’s is an instinctive information vault that’s indigenous to songwriters, a little box you can go to and all of your songs are in there. An imaginary barrel - that’s what I always used to call it.” (Bee Gees, n.d.)
9 Bruce Springsteen: Trying to describe the creative process, Springsteen says: “When you are writing well, you are not exactly sure how you have done it, or if you’ll ever do it again. You’re looking for the element you cannot explain. The element that breathes life and character into the people or situation you are writing about. … So to do that, you gotta tap something more than … well, it cannot just be math. There’s got to be some mystical aspect to it. And when that third element arrives, it’s sort of one and one makes three” (Carlin, 2012, p. 72).
10 Paul Simon: *Writing good music is like plugging into the universe.* Reflecting on his writing of the epic “Sounds of Silence”: “I was really too young to know that there are times when—I do not want to sound silly—but when you are plugged into the universe and all of a sudden something comes through you, and it’s yours but it is not yours. … It comes out and you do not know where it comes from. I do not know why or how I wrote that song when I was 21 or 22 years old. It was certainly beyond me.” (Gonzales, 2016)

### 4.4 The Big-C narrative

**Figure 2:**
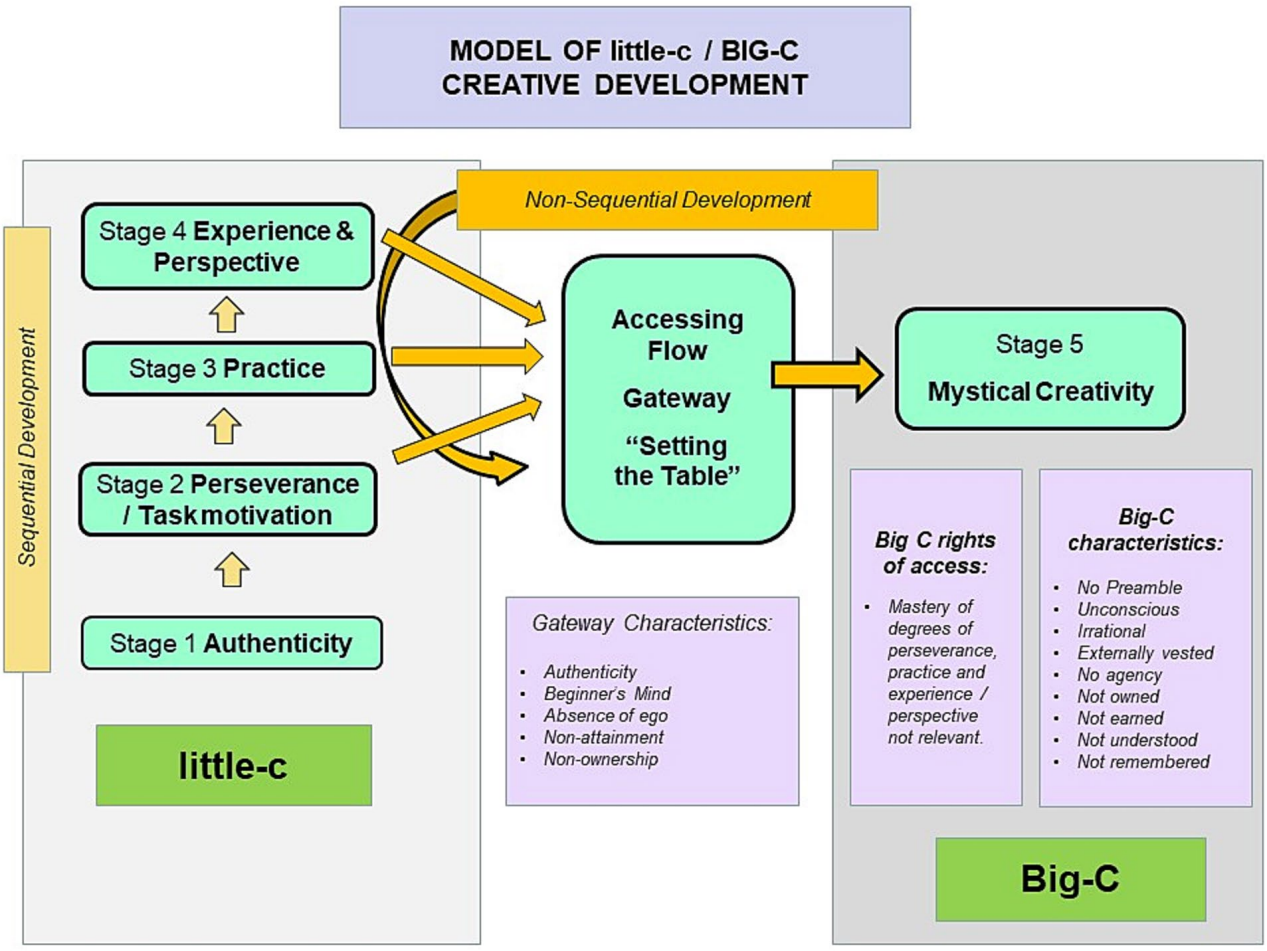
The little-c/Big-C model of creative development. Note that while little-c is shown to develop sequentially through stages 1 to 4, in contradistinction, the attainment of Big-C is not sequentially path-dependent. Presuming sufficient authenticity and perseverance/task motivation (stages 1 and 2), degrees of practice (stage 3) and experience (stage 4) become less relevant in accessing Big-C whose characteristics map with those of Big-P.

Similar to the Big-P phenomenon, from the evidence above we can infer that that Big-C moments are equally rare, transient and random. Paraphrasing from Amabile (1983), Simonton (2014), and Priilaid and Callaghan (2023), we suggest that with sufficient perseverance and task motivation, Big-C moments may occur non-sequentially with var*ying* levels of expertise and experience; both high and low. Within this frame, committed novices like a 21-year old Paul Simon stand as much chance of producing moments of creative genius (like “Sounds of Silence”) as wizened masters like a 78-year old Bob Dylan (when he wrote “Murder most Foul”). As with Cassian-Merton, our findings indicate that Big-C moments are seldom if ever owned, or rationally understood. Likewise, their preconditions are also similar to those of Big-P. Both involve table-setting: being in the moment, staying true to one’s self, shedding ego and letting go. Setting the table becomes thus a portal, a magical Narnia cupboard, though with no guarantee of automatic entry. Figure 2 depicts the little-c/Big-C dynamic as per above.

## 5 Discussion

He remembered her saying: *I do not believe artists know half the time what they are creating. Oh yes, all the tralala, the technique – that’s another matter. But like ordinary people who get out of bed, wash their faces, comb their hair, cut the tops off their boiled eggs, they do not act, they are instruments which are played on, or vessels which are filled – in many cases only with longing.* Was it this? or had he dreamed or imagined, or heard it from another quarter?

Patrick White, *The Vivisector.*

Going to the deepest level of communication,

Where back and forth has never stopped.

Where I am not the initiator but the transmission wire itself.

Rohr (2024).

While a singular study may not be enough to firmly establish a thesis, in this particular work, by drawing upon the cumulative evidence from multiple cases, we offer an insightful perspective on how numerous artists experience profound creativity. Within the sample of the artists observed, we note how they have employed the same language to both describe their Big-C experiences and to distinguish between these and more common moments of little-c creativity. So doing, we conclude that there is indeed a difference between the two. By contrast, in 2014 Runco described the Big-C/little-c construct as a false dichotomy, arguing that “the processes involved in everyday creativity are the same as those involved in high end creative achievements” (Runco, 2014, p. 131). Based on the evidence presented in our study, we have reached a contrasting conclusion. We find that in terms of character and development, little-c and Big-C are indeed distinct and dichotomous. This statement requires some qualification however; for those finding themselves in the Big-C proceed in good faith *from* the disposition of little-c. At the top end of the little-c continuum, we argue that all creatives are aiming to produce creditable output and work from the *same* suite of technical options. Even controlling for degrees of perseverance, practice and experience, in truth it seems that they can do no more. There is nothing, it seems, that one can do to earn entry to Big-C. For song-writers, Big-C is the momentary access to an upland which no one conquers, to paraphrase Leonard Cohen. Those that gain access do so as absolute beginners, by *not* trying, by *not* controlling through the application of any imagined method or formula. For Dan Fogelberg, songwriting was something deeply counter-intuitive, stating: “I do it and I do not understand it. It’s just so amazingly unconscious. It’s the most *mystical* thing I’ve ever experienced” (Zollo, 2021). At this point of our understanding, Fogelberg’s conclusion is perhaps the most we can say of Big-C: that like Big-P, deeply creative songwriting is indeed a mystical phenomenon that we can characterise, but not explain. As such the practice of Big-C has attracted shibboleth notions of “invoking the muse,” of possessing the “magic touch,” of meriting “divine inspiration,” and finding moments of “serendipity.” These are terms that we reach for in the absence of anything to explain the mystery that it happens randomly, and often irrespective of age and experience.

Within in this context it is worth returning to the bigger issue of Mysticism and its relation to creativity. Tracking its historical development, Schmidt (2003) argues that Mysticism has evolved through various sea changes, commencing from a specifically Christian position, to one which, by 1900, had become universalist in character. This is best captured by William James, who, in 1901, put it that “the mother sea and fountain head of all religions lies in the mystical experiences of the individual, taking the word mystical in a very wide sense” (James, 2001, p. 501). In line with James’s liberal-unitarian perspective and recognizing the kinship between Big-P and Big-C, which both draw from the same mystical source, we posit tentatively that Big-C could be regarded as a third wave of Mysticism. Derived from the Big-C experiences cited in this paper, this third wave positioning suggests that Big-C serves as an equivalent means of connecting with the Mystic.

While so observing the profoundly mystical character of both Big-P and Big-C, we believe however that they are probably not identical phenomena. In this sense what we are more likely looking at is a two-set Venn diagram depicting overlapping similarities, the extent of which, for now, remains scientifically unclear. And this is a frontier of psychology to be explored. One approach here lies in neuropsychology (see Mastria et al., 2021; Ovando-Tellez et al., 2022), which seeks to integrate psychoanalytic models with the insights of modern neuroscience in order to unravel the neural and psychological underpinnings of such experiences. A further applied extension of our findings would be to discover what neurological triggers assist in clearing the barriers of ego and agency towards a less self-conscious version of creativity. What kind of scientific intervention might enable this in art? in sport? in work? in elevating the mundane moments of the creative dog-day? This represents a frontier opportunity for the application of rigorous scientific endeavour. That said, as per Big-C descriptions offered by the songwriters in this study, we caution however that profound creativity is likely to resist attempts to domesticate, commoditize, monetize and, in today’s age of AI, automate it. As with Big-P, moments of Big-C in song writing appear to be achieved when exactly the opposite occurs.

## 6 Summary and conclusion

To summarise our study: the progression towards achieving “little-c” creativity can be characterized as following a somewhat predetermined path. It begins with an emotional trigger, leading to the development of perseverance and regular practice. This, in turn, leads to a level of expertise, from which perspective, unique expression, and professional mastery ultimately arise. Given this developmental path, our research was motivated to explore the differences between “little-c” and “Big-C” moments of creativity, especially how and why instances of “Big-C” creativity occur as randomly as they do. To initiate this study we formalize the Cassian-Merton model of prayer development where we liken little-p to conventional prayer and Big-P to mystical prayer. So doing, we compare the developmental paths of prayer to subjective accounts of creativity in famous Anglophone artist-musicians from the period 1960 to 1980. Our main findings reveal a symmetry between the development of prayer and creativity. “Little-c” creativity follows a sequential path, whereas “Big-C” creativity can bypass extensive practice and experience, emerging spontaneously with a sense of agency surrender.

In conclusion, we note that these findings are specific to songwriting and may not easily extend to other creative fields, despite any related aspects. We would be interested in comparative studies in domains of science and technology as well as longer creative projects such as novel writing.

## Data Availability

The original contributions presented in the study are included in the article/supplementary material, further inquiries can be directed to the corresponding author.

## Footnotes

^1^We acknowledge that much of the historical work on prayer was recorded in Latin or ancient Greek and there might well be such texts that have as yet not been translated into English.

## References

[ref1] Acar S. Runco M. A. (2019). Divergent thinking: new methods, recent research, and extended theory. Psychol. Aesthet. Creat. Arts 13, 153–158. doi: 10.1037/aca0000231

[ref2] Amabile T. M. (1983). The social psychology of creativity: a componential conceptualization. J. Pers. Soc. Psychol. 45, 357–376. doi: 10.1037/0022-3514.45.2.357

[ref3] Amabile T. (2011). Componential theory of creativity. Boston, MA: Harvard Business School, 538–559.

[ref4] Andreasen N. C. (1987). Creativity and mental illness: prevalence rates in writers and their first-degree relatives. Am. J. Psychiatry 144, 1288–1292. doi: 10.1176/ajp.144.10.1288, PMID: 3499088

[ref5] Baas M. Nijstad B. A. Boot N. C. De Dreu C. K. (2016). Mad genius revisited: vulnerability to psychopathology, biobehavioral approach-avoidance, and creativity. Psychol. Bull. 142, 668–692. doi: 10.1037/bul0000049, PMID: 26950008

[ref6] Beatles Bible. #9 Dream. (2010). Accessed December 11, 2020. Available at: https://www.beatlesbible.com/people/john-lennon/songs/9-dream/; En.beatlesbible.com

[ref7] Bee Gees. Quotes: What the Bee Gees have said about… (n.d.). Accessed 12 December 2020. Available at: https://www.beegees-world.com/quotes.html

[ref8] Beghetto R. A. Kaufman J. C. (2007). Toward a broader conception of creativity: a case for" mini-c" creativity. Psychol. Aesthet. Creat. Arts 1, 73–79. doi: 10.1037/1931-3896.1.2.73

[ref9] Benedek M. Borovnjak B. Neubauer A. C. Kruse-Weber S. (2014). Creativity and personality in classical, jazz and folk musicians. Personal. Individ. Differ. 63, 117–121. doi: 10.1016/j.paid.2014.01.064, PMID: 24895472 PMC3989052

[ref10] Campbell D. T. (1960). Blind variation and selective retention in creative thoughts in other knowledge processes. Psychol. Rev. 67, 380–400. doi: 10.1037/h0040373, PMID: 13690223

[ref11] Carlile P. R. Christensen C. M. (2005). The cycles of theory building in management research. Cambridge, MA: Division of Research, Harvard Business School.

[ref12] Carlin P. A. (2012). Bruce. London: Simon & Schuster, 424.

[ref13] Cavrell L. (1966). The Universal Mind of Bill Evans [Film]. Rhapsody Films.

[ref14] Cocker J. (2012). Leonard Cohen to Jarvis Cocker: ‘I’ve always felt I was scraping the bottom of the barrel’. NME. Available at: https://www.nme.com/news/music/leonard-cohen-24-1274530

[ref15] Cohen L. (2011). How I got my song: Prince of Asturias awards [video recording]. YouTube. Available at: https://www.youtube.com/watch?v=VIR5ps8usuo

[ref16] Cross C. R. (2005). The Beatles: Day-by-Day, Song-by-Song, Record-by-Record. London: Lincoln, 464–465.

[ref7001] Crosby D. Stills S. Nash G. Young N. (1970). Déjà Vu [Album]. Atlantic Records.

[ref17] Cunningham L. (1999). Thomas Merton and the monastic vision. William B. Eerdmans Publishing Company, Cambridge.

[ref18] Dávila C. M. Ruiz F. O. (2019). Subjects analysing subjects in the biographical approach: a generational study of Chilean musicians. Contemp. Soc. Sci. 14, 463–474. doi: 10.1080/21582041.2018.1448939

[ref19] Dehaene S. Lau H. Kouider S. (2021). What is consciousness, and could machines have it? Robot. AI. Human., 43–56. doi: 10.1007/978-3-030-54173-6_4 29074769

[ref20] Eliot M. (2010). Paul Simon: a life. New York, NY: John Wiley & Sons.

[ref21] Ellwood A. (2013). History of the eagles [Flim]. [DVD] AV Channel.

[ref22] Evans B. (1959). “Kind of Blue [CD]” in Linear Notes.

[ref23] Feist G. J. (1998). A meta-analysis of personality in scientific and artistic creativity. Personal. Soc. Psychol. Bull. 2, 290–309. doi: 10.1207/s15327957pspr0204_5 15647135

[ref24] Feist G. J. (2010). “The function of personality in creativity: the nature and nurture of the creative personality” in The Cambridge handbook of creativity. eds. Kaufman J. C. Sternberg R. J. (New York, NY: Cambridge University Press), 113–130.

[ref25] Feist G. J. (2019). Creativity and the big two model of personality: plasticity and stability. Curr. Opin. Behav. Sci. 27, 31–35. doi: 10.1016/j.cobeha.2018.07.005

[ref26] Feldman D. H. Csikszentmihalyi M. Gardner H. (1994). Changing the world: A framework for the study of creativity. Westport, CT: Praeger.

[ref27] Ferrarotti F. (2005). On the science of uncertainty: The biographical method in social research. Lanham, MD: Lexington Books.

[ref28] Forgeard M. (2024). Creativity and resilience: creativity from, or through adversity? Creat. Res. J., 1–8. doi: 10.1080/10400419.2023.2299639

[ref29] Frey G. (2015). Glenn Frey on the Dan Patrick show (part 1) [video]. YouTube. Available at: https://www.youtube.com/watch?v=kUcncHXnlVE.

[ref30] Fürst G. Ghisletta P. Lubart T. (2017). An experimental study of the creative process in writing. Psychol. Aesthet. Creat. Arts 11, 202–215. doi: 10.1037/aca0000106

[ref31] Gardner H. (2011). Creating minds: An anatomy of creativity seen through the lives of Freud, Einstein, Picasso, Stravinsky, Eliot, Graham, and Ghandi. London: Civitas Books.

[ref32] Gonzales S. (2016). Songwriter Paul Simon speaks about beauty and the ‘infinity’ of pleasurable pursuits. Yale News. Retrieved September 13, 2016. Available at: http://news.yale.edu/2016/04/06/songwriter-paul-simon-speaks-about-beauty-and-infinity-pleasurable-pursuits

[ref33] Hadamard J. (1945). An essay on the psychology of invention in the mathematical field. Princeton, NJ: Princeton University Press.

[ref34] Harmless W. (2004). Desert Christians: An introduction to the literature of early monasticism. Oxford, NY: Oxford University Press.

[ref36] Isaacson W. (2011). Steve Jobs. London: Little-Brown, 415–416.

[ref37] James W. (1902). The varieties of religious experience: a study in human nature. London: Longmans, Green and Co.

[ref38] James W. (2001). The correspondence of William James, July 1899–1901. Charlottesville: University Press of Virginia.

[ref39] Kaufman J. C. Beghetto R. A. (2009). Beyond big and little: the four c model of creativity. Rev. Gen. Psychol. 13, 1–12. doi: 10.1037/a0013688

[ref40] Kozbelt A. Beghetto R. A. Runco M. A. (2010). “Theories of creativity” in The Cambridge handbook of creativity. eds. Kaufman J. C. Sternberg R. J. (New York, NY: Cambridge University Press), 20–47.

[ref41] Lord C. (2024). The reader interview. David Gilmour: ‘the rich and powerful have siphoned off the majority of music industry money’. Guardian. Retrieved October 28, 2024. Available at: https://www.theguardian.com/music/2024/oct/03/david-gilmour-the-rich-and-powerful-have-siphoned-off-the-majority-of-music-industry-money

[ref42] Lubart T. I. (2001). Models of the creative process: past, present and future. Creat. Res. J. 13, 295–308. doi: 10.1207/S15326934CRJ1334_07

[ref43] Ludwig A. M. (1992). Creative achievement and psychopathology: comparison among professions. Am. J. Psychother. 46, 330–354. doi: 10.1176/appi.psychotherapy.1992.46.3.330 1530096

[ref44] Macatee R. (2016). David bowie talks about “searching for god” and “spirituality” in unaired 60 minutes interview. E! Online. Retrieved October 13, 2020. Available at: https://www.eonline.com/news/730566/david-bowie-talks-about-searching-for-god-and-spirituality-in-unaired-60-minutes-interview

[ref45] Mastria S. Agnoli S. Zanon M. Acar S. Runco M. A. Corazza G. E. (2021). Clustering and switching in divergent thinking: neurophysiological correlates underlying flexibility during idea generation. Neuropsychologia 158:107890. doi: 10.1016/j.neuropsychologia.2021.107890, PMID: 34010602

[ref46] McDonough J. (2002a). Shakey: Neil Young’s biography. London: Random House.

[ref47] McDonough J. (2002b). Shakey: Neil Young’s Biography. New York, NY: Random House, 127.

[ref48] McGinn B. (1991). The foundations of mysticism: Origins to the fifth century. New York, NY: Crossroads.

[ref49] Merrotsy P. (2013). A note on big C creativity and little-c creativity. Creat. Res. J. 25, 474–476. doi: 10.1080/10400419.2013.843921

[ref50] Merton T. (1948). The seven storey mountain. New York, NY: Garden City Books.

[ref51] Merton T. (2012). Thomas Merton on contemplation (unabridged). [audio CD]. Anthony Ciorra (introduction). Rockville, MD: Now You Know Media.

[ref52] Miles B. (1997). Paul McCartney: Many Years From Now. New York, NY: Henry Holt & Company, 201–202.

[ref53] Montandon M. (Ed.) (2007). Innocent When You Dream: Tom Waits: The Collected Interviews. (p. 155). London: Orion Books.

[ref54] Nakamura J. Csikszentmihalyi M. (2002). “The concept of flow” in Handbook of positive psychology. eds. Snyder C. R. Lopez S. J. (Oxford, England: Oxford University Press), 89–105.

[ref55] Nijstad B. A. Stroebe W. (2006). How the group affects the mind: a cognitive model of idea generation in groups. Personal. Soc. Psychol. Rev. 10, 186–213. doi: 10.1207/s15327957pspr1003_1, PMID: 16859437

[ref56] Ovando-Tellez M. Benedek M. Kenett Y. N. Hills T. Bouanane S. Bernard M.. (2022). An investigation of the cognitive and neural correlates of semantic memory search related to creative ability. Commun. Biol. 5:604. doi: 10.1038/s42003-022-03547-x, PMID: 35710948 PMC9203494

[ref57] Post F. (1994). Creativity and psychopathology a study of 291 world-famous men. Br. J. Psychiatry 165, 22–34. doi: 10.1192/bjp.165.1.22, PMID: 7953036

[ref58] Priilaid D. (2023). Creativity explained – From music and art to innovation in business. Cape Town: Press. Open Access.

[ref59] Priilaid D. Callaghan C. (2023). The creative development process: biographical insights on innovation in Anglophone music. Acta Commercii 23, 1–15. doi: 10.4102/ac.v23i1.1129

[ref60] Rachel D. (2013). Isle of noises – Conversations with great British songwriters. London: Picador.

[ref61] Ratzinger C. J. (2004). Introduction to Christianity: yesterday, today and tomorrow. Commun. Int. Catholic Rev. 31, 481–495.

[ref62] Richards K. (2010). Life. London: Weidenfeld & Nicolson, 182.

[ref63] Ricks C. (2003). Dylan’s Visions of Sin. London: Penguin Books, 47.

[ref65] Rohr R. (2003). Everything belongs: The gift of contemplative prayer. New York, NY: Crossroad Publ.

[ref66] Rohr R. (2015). Desert Christianity and the eastern fathers of the church, the mendicant, 5(2) (march 2015), 1.

[ref67] Rohr R. (2023). Richard Rohr’s daily meditation. The Centre for Action and Contemplation.

[ref68] Rohr R. (2024). Richard Rohr’s daily meditation. The Centre for Action and Contemplation.

[ref69] Rossman J. (1931). The motives of inventors. Q. J. Econ. 45, 522–528. doi: 10.2307/1883903

[ref70] Runco M. A. (2014). “Big C, little c” creativity as a false dichotomy: reality is not categorical. Creat. Res. J. 26, 131–132. doi: 10.1080/10400419.2014.873676

[ref71] Runtagh J. (2018). Graham Nash Tells the Wild Tales Behind His Most Enduring Songs. Retrieved 11 January 2021. Available at: https://jonimitchell.com/library./print.cfm?id=4187

[ref73] Schmidt L. E. (2003). The making of modern mysticism. J. Am. Acad. Relig. 7, 273–302.

[ref74] Scorsese M. (2019). Rolling Thunder Revue: A Bob Dylan Story. Ditto: [DVD] AV Channel.

[ref75] Settecase P. (2024). Can we think machines are conscious? A survey of philosophical problems facing the attribution of consciousness to machines. J. Artif. Intell. Conscious. 11, 35–50. doi: 10.1142/S2705078524500073

[ref76] Sica C. Novara C. Dorz S. Sanavio E. (1997). Coping strategies: evidence for cross-cultural differences? A preliminary study with the Italian version of coping orientations to problems experienced (COPE). Personal. Individ. Differ. 23, 1025–1029. doi: 10.1016/S0191-8869(97)00112-8

[ref77] Simonton D. K. (1999). Creativity as blind variation and selective retention. Is the creative process Darwinian? Psychol. Enquiry 10, 309–328.

[ref78] Simonton D. K. (2000). Creative development as acquired expertise: theoretical issues and an empirical test. Dev. Rev. 20, 283–318. doi: 10.1006/drev.1999.0504

[ref79] Simonton D. K. (2014). Creative perfomance, expertise acquisition, individual differences and developmental antecedents: an integrative research agenda. Intelligence 45, 66–73. doi: 10.1016/j.intell.2013.04.007

[ref80] Simpson D. (2018). Simply Red: how we made Holding Back the Years. The Guardian. (Accessed December 11, 2020). Available at: https://www.theguardian.com/music/2018/nov/27/simply-red-how-we-made-holding-back-the-years-mick-hucknall

[ref81] Simpson D. (2019a). Keane: how we made Somewhere Only We Know. The Guardian. Retrieved November 15, 2019. Available at: https://www.theguardian.com/culture/2019/sep/17/how-we-made-keane-somewhere-only-we-know-tom-chaplin-time-rice-oxley

[ref82] Simpson D. (2019b). Lloyd Cole and the Commotions: how we made Rattlesnakes. The Guardian. Accessed December 11, 2020. Available at: https://www.theguardian.com/music/2019/oct/08/lloyd-cole-and-the-commotions-how-we-made-rattlesnakes

[ref83] Sounes H. (2011). Down the highway: the life of bob Dylan. New York, NY: Doubleday, 230.

[ref84] Stein M. I. (1953). Creativity and culture. J. Psychol. 36, 311–322. doi: 10.1080/00223980.1953.9712897

[ref85] Suzuki S. (2020). Zen mind, beginner’s mind. 50th anniversary Edn. Boston, MA: Shambhala.

[ref86] Sweet B. (1994). Steely Dan – Reelin’ in the years. London: Omnibus Press.

[ref87] Taylor J. (2011). Interview extract – quoted off interview on the Charlie Rose show [Video recording]. Available at: https://charlierose.com/videos/14497

[ref88] Torrance E. P. (1988). “The nature of creativity as manifest in its testing” in The nature of creativity: Contemporary psychological perspectives. ed. Sternberg R. J. (New York, NY: Cambridge University Press), 43–75.

[ref89] Torrance E. P. (1995). Why fly? Westport, CT: Greenwood Publishing Group.

[ref90] Treffinger D. J. (1995). Creative problem solving: overview and educational implications. Educ. Psychol. Rev. 7, 301–312. doi: 10.1007/BF02213375

[ref91] Turner G. (1962). Bob Dylan: A New Voice Singing New Songs. Sing Out Magazine, 1–7.

[ref92] Underhill E. (1910). Mysticism: A study in nature and development of spiritual consciousness. New York, NY: Dutton.

[ref93] Wallas G. (1926). The art of thought. New York, NY: Harcourt.

[ref94] Watts L. L. Steele L. M. Medeiros K. E. Mumford M. D. (2019). Minding the gap between generation and implementation: effects of idea source, goals, and climate on selecting and refining creative ideas. Psychol. Aesthet. Creat. Arts 13, 2–14. doi: 10.1037/aca0000157

[ref95] Wegman J. (2000). The Story Behind ‘Purple Haze’. npr.org. (Accessed December 11, 2020). Available at: https://www.npr.org/2000/09/18/1088122/jimi-hendrix-purple-haze

[ref96] Young N. (2013). Waging Heavy Peace: A Hippie Dream. New York, NY: Plume, 344.

[ref97] Zollo P. (2021). Behind the song: Dan Fogelberg, “to the morning’. Retrieved October 28, 2024. Available at: https://americansongwriter.com/todays-song-for-12-16-2020-dan-fogelberg-to-the-morning/

